# Acoelomorph flatworm monophyly is a long-branch attraction artefact obscuring a clade of Acoela and Xenoturbellida

**DOI:** 10.1098/rspb.2024.0329

**Published:** 2024-09-18

**Authors:** Anthony K. Redmond

**Affiliations:** ^1^ School of Medicine, University College Dublin, Dublin, Ireland

**Keywords:** Acoelomorpha, Xenacoela, *Xenoturbella*, Xenacoelomorpha, bilaterian phylogenomics, long-branch attraction

## Abstract

Acoelomorpha is a broadly accepted clade of bilaterian animals made up of the fast-evolving, morphologically simple, mainly marine flatworm lineages Acoela and Nemertodermatida. Phylogenomic studies support Acoelomorpha’s close relationship with the slowly evolving and similarly simplistic *Xenoturbella*, together forming the phylum Xenacoelomorpha. The phylogenetic placement of Xenacoelomorpha amongst bilaterians is controversial, with some studies supporting Xenacoelomorpha as the sister group to all other bilaterians, implying that their simplicity may be representative of early bilaterians. Others propose that this placement is an error resulting from the fast-evolving Acoelomorpha, and instead suggest that they are the degenerate sister group to Ambulacraria. Perhaps as a result of this debate, internal xenacoelomorph relationships have been somewhat overlooked at a phylogenomic scale. Here, I employ a highly targeted approach to detect and overcome possible phylogenomic error in the relationship between *Xenoturbella* and the fast-evolving acoelomorph flatworms. The results indicate that the subphylum Acoelomorpha is a long-branch attraction artefact obscuring a previously undiscovered clade comprising *Xenoturbella* and Acoela, which I name Xenacoela. The findings also suggest that Xenacoelomorpha is not the sister group to all other bilaterians. This study provides a template for future efforts aimed at discovering and correcting unrecognized long-branch attraction artefacts throughout the tree of life.

## 1. Background

Xenacoelomorpha is an enigmatic, typically marine, phylum of invertebrate bilaterian animals [1–4]. They are characterized by apparently simple morphology, particularly their acoelomate body plan and the absence of nephridia, but also lack characteristic features found in many bilaterians such as a through-gut, circulatory and respiratory systems, and a complex brain [1,2,4]. However, recent studies focused on the lineage have revealed a remarkable diversity in nervous system morphology [5–7], as well as evidence for active excretion despite the lack of a specialized organ [8,9], implying underappreciated biological complexity in these species. The lineage is divided into two subphyla [10]; the fast-evolving Acoelomorpha [11], consisting of the two acoelomorph flatworm classes Acoela and Nemertodermatida, and the more slowly evolving Xenoturbellida [3,12], from which only the genus *Xenoturbella* is known.

**Figure 1.**
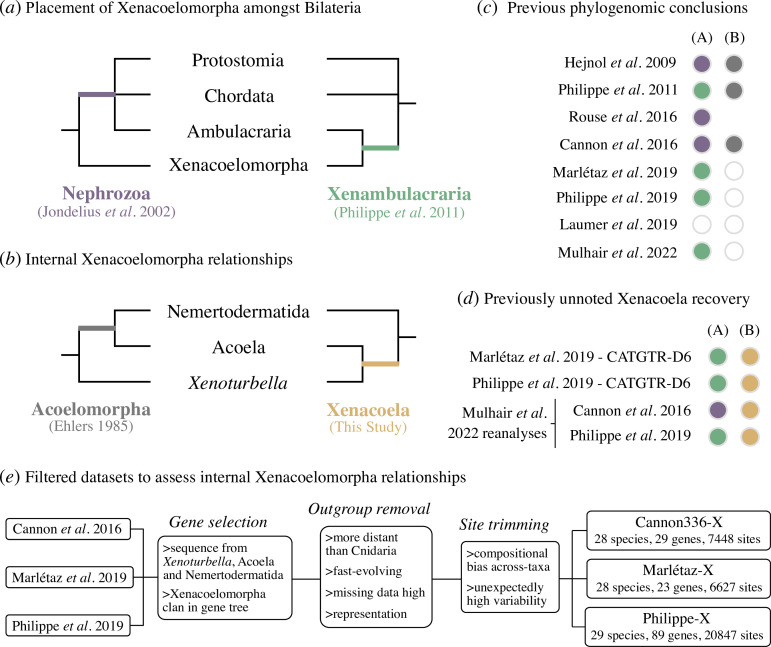
Hypothesized Xenacoelomorpha relationships and dataset preparation. (*a*) Conflicting hypotheses for Xenacoelomorpha’s place within Bilateria as either the sister group to all other bilaterians (Nephrozoa; purple) or Ambulacraria (Xenambulacraria; green). Chordata, Protostomia and (Xen)Ambulacraria are shown as a polytomy because deuterostome monophyly has been disputed. (*b*) Internal Xenacoelomorpha relationships: Acoelomorpha (grey), the generally accepted hypothesis, as compared with the newly proposed Xenacoela (gold). (*c*) Conclusions from past phylogenomic studies are shown with the relationship in question and colours based on (*a*) and (*b*). White/empty circles indicate relationships that were not discussed in that study (though it is noteworthy that all Laumer *et al.* [27] topologies show Nephrozoa and Acoelomorpha), while the lack of a circle for Rouse *et al*. [25] indicates that taxon sampling precluded assessment of support for Acoelomorpha/Xenacoela. (*d*) Unreported recovery of Xenacoela in key past analyses with the relationship in question and colours based on (*a*) and (*b*). (*e*) Filtering approach used to produce error-avoidant, internal Xenacoelomorpha-targeted datasets.

Although morphological similarity has long been noted between *Xenoturbella* and Acoelomorpha [13–16], their confident joining within Xenacoelomorpha is a relatively recent phylogenomic discovery [3,17–19]. While the original assignment of these lineages as ‘turbellarian’ flatworms has been rejected alongside any close relationship to Platyhelminthes [3,4,11,15,17,18,20–24], consensus on the placement of Xenacoelomorpha within Bilateria has not been reached. Some studies favour Xenacoelomorpha as the sister group to all other bilaterians (‘Nephrozoa’) [17,18,25,26], which could potentially indicate that their apparently simple morphology may represent the early bilaterian state (figure 1*a*), while others argue that Xenacoelomorpha is the sister group to Ambulacraria (‘Xenambulacraria’) [3,28–30], which could be more consistent with their simplicity being degenerate (figure 1*a*). Proponents of Xenambulacraria implicate Nephrozoa to be a systematic error induced by the fast-evolving and long-branching Acoelomorpha [3,28–32]. Other placements have also been recovered, such as sister to either deuterostomes [3] or protostomes [25], while some efforts to resolve this problem have even questioned the monophyly of deuterostomes [28,33].

This ongoing problem highlights the importance of the underlying phylogenomic methodology. Past studies have shown that compositional heterogeneities across sites and taxa are important biasing factors in resolving bilaterian relationships [28,29,31,34]. Strategies to reduce such heterogeneity include the use of the GTR (a general time reversible amino acid exchangability matrix inferred from the data) + CAT (a mixture of equilibrium frequency categories/classes inferred from the data) model [35], which accommodates heterogeneity across sites [34,35], and the recoding of amino acids into smaller alphabets based on their evolutionary or biochemical properties (e.g. the six Dayhoff categories [36]), which can reduce heterogeneity across both sites and taxa, but risks masking informative substitutions [37–42]. Hidden paralogy, where non-orthologous genes are unintentionally incorporated into phylogenomic datasets, and other data errors can also mislead phylogenomic inference [29,43,44]. Recent studies suggest that such data and modelling errors bias phylogenomic results towards Nephrozoa, and that Xenambulacraria is supported when minimizing the impact of hidden paralogy and compositional heterogeneity [28–32].

Given their importance for understanding bilaterian evolution, resolving their placement among other animals has understandably been the main goal of phylogenomic analyses including Xenacoelomorpha [18,25,28,29,31]. Combined with studies generally and expectedly recovering monophyletic Acoela, Nemertodermatida, Acoelomorpha and *Xenoturbella* [3,18], and the paucity of genome data for the lineage (though see [9,28,45–47]), this has resulted in little focus on internal xenacoelomorph relationships. Here, using both empirical and simulation approaches intended to detect and overcome phylogenomic error, I reassess the relationships between *Xenoturbella* and the fast-evolving acoelomorph flatworms. I conclude that Acoelomorpha is a long-branch attraction artefact obscuring a clade comprising *Xenoturbella* and Acoela, which I tentatively name ‘Xenacoela’ (figure 1*b*). Furthermore, the results are not consistent with the Nephrozoa hypothesis of bilaterian evolution.

## 2. Results

### (a) Unacknowledged support for Xenacoela in past studies

Since its formal proposal by Philippe *et al.* [3], only two studies, which focused primarily on Xenacoelomorpha’s placement within Bilateria, have generated large-scale phylogenomic datasets and have also included members of the three major lineages: Cannon *et al*. [18] recovering Nephrozoa, and Philippe *et al*. [28] recovering Xenambulacraria. Others exclude Nemertodermatida [25] or employ pre-existing datasets [29]. However, off-target studies, such as those of Marlétaz *et al*. [31] and Laumer *et al.* [27], sometimes include all three xenacoelomorph lineages. To reassess the relationships between *Xenoturbella* and the acoelomorph flatworms, I surveyed the phylogenies produced in these past studies. While most support the expected sister group relationship between *Xenoturbella* and Acoelomorpha, including all analyses in Cannon *et al*. [18], more recent studies did not address internal Xenacoelomorpha relationships [28,29,31] (figure 1*c*). However, key analyses in these studies have sometimes recovered the alternative Xenacoela clade proposed here (figure 1*d*), particularly when the best efforts to minimize phylogenetic error are made. Specifically, GTR + CAT analyses in Philippe *et al*. [28] did not recover strong support for Acoelomorpha, while combining GTR + CAT with recoding recovered strong support for Xenacoela (figure 1*d*). Philippe *et al*. [28] also reanalysed the Cannon *et al*. [18] dataset using GTR + CAT with recoding and did not recover strong support for Acoelomorpha (figure 1*d*). Marlétaz *et al*. [31], although focused on spiralian phylogeny, also recovered Xenacoela when combining GTR + CAT with recoding (figure 1*d*), while Mulhair *et al*. [29] recovered Xenacoela without recoding in reanalyses of the Cannon *et al*. [18] and Philippe *et al*. [28] datasets when minimizing hidden paralogy (figure 1*d*). This recovery across multiple studies when attempting to avoid phylogenetic error indicates that Xenacoela warrants consideration as an alternative to Acoelomorpha.

### (b) Filtering datasets to reduce error and target internal xenacoelomorph relationships

To better understand the source of the signal for Xenacoela compared with Acoelomorpha, I reanalysed datasets from past studies, taking special care to minimize phylogenetic error in resolving internal xenacoelomorph relationships (figure 1*e*). I filtered these datasets to only retain genes that: (i) had at least one representative from each of the three major lineages (*Xenoturbella*, Acoela and Nemertodermatida) and (ii) where Xenacoelomorpha could be recovered as a clan (the equivalent of a ‘monophyletic’ group but in an unrooted tree [48]) in gene trees, resulting in the exclusion of many genes where Xenacoelomorpha is potentially affected by hidden paralogy (a known issue in placing the lineage amongst bilaterians [28,29,31]) or limited/biased phylogenetic signal (figure 1*e*). Approximately unbiased (AU) topology tests [49] indicate that this filtering process enriches for genes that do not reject Xenacoela (electronic supplementary material, figure S1), suggesting that at least some support for Acoelomorpha is associated with genes that do not recover Xenacoelomorpha.

Bilaterian phylogenomics is known to be susceptible to inadequate phylogenetic modelling and systematic errors [28–31,33,34]. As distant outgroups can worsen these issues and produce incorrect topologies [50,51], I removed outgroups more distant than Cnidaria and subsampled off-target bilaterian species to balance outgroup representation and exclude fast-evolving species with high levels of missing data (figure 1*e*). Alignment sites that either contribute to across taxa compositional heterogeneity or have unusually high variability were also trimmed [52] (figure 1*e*).

This filtering approach was applied to three key datasets: the 336 gene ‘best sampled taxa’ dataset from Cannon *et al*. [18], the least saturated genes dataset used in the main analyses of Marlétaz *et al*. [31] and the main 1173 gene dataset from Philippe *et al*. [28]. After filtering, this resulted in the following new datasets: Cannon336-X (‘X’ for Xenacoelomorpha) with 29 genes, 7448 sites and 28 taxa; Marlétaz-X with 23 genes, 6627 sites and 28 taxa, and Philippe-X with 89 genes, 20 847 sites and 29 taxa (figure 1*e*). The resultant datasets are notably smaller than those in the original studies, but are comparable to those used by Mulhair *et al*. [29] to reassess Xenacoelomorpha’s placement amongst Bilateria, and should have substantially improved signal-to-noise ratios for internal xenacoelomorph relationships. Additional details on dataset selection and filtering are in electronic supplementary material, methods subsection ‘Dataset Preparation’.

### (c) Improved model fit correlates with support for Xenacoela over Acoelomorpha

**Figure 2.**
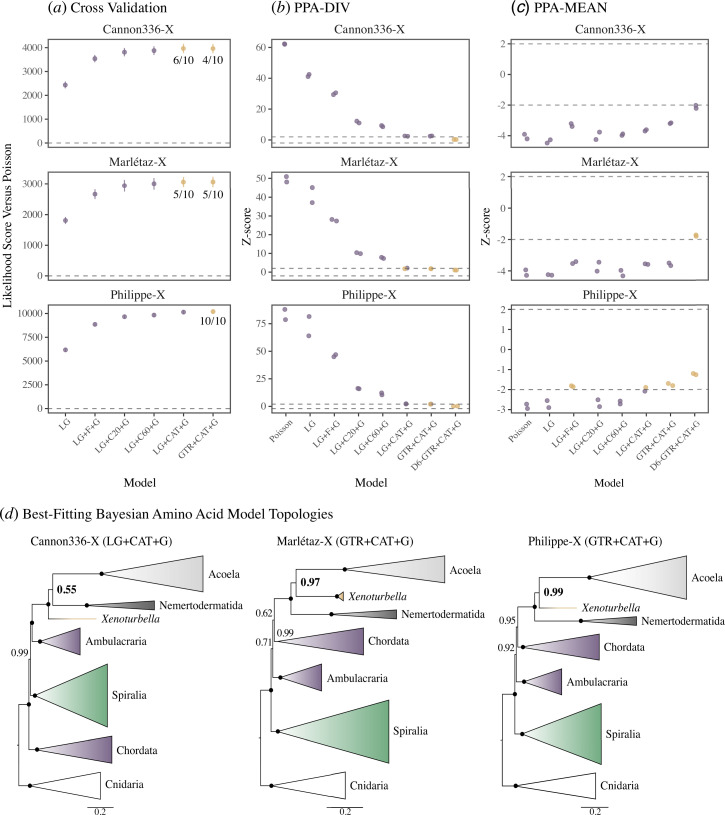
Phylobayes Bayesian model fit and phylogenomic analyses of the Cannon336-X, Marlétaz-X and Philippe-X datasets**.** (*a*) Tenfold cross-validation test of relative model fit for each dataset. Gold data points represent models included in the set of best-fitting models (excluded models are in purple), with a value out of 10 representing how often this model fits best in test replicates. PPA test Z-scores for each dataset and modelling strategy for the (*b*) PPA-DIV and (*c*) PPA-MEAN tests with results from two Phylobayes chains each shown. Dashed vertical lines at |Z| = 2 and |Z|=−2 indicate pass/fail interpretation. (*d*) Topologies for each dataset under the best-fitting models (LG + CAT + G shown for Cannon336-X although GTR + CAT + G fits similarly well; GTR + CAT + G shown for Marlétaz-X based on better PPA results than LG +CAT + G) are shown with species collapsed into major clades. Posterior probabilities are shown for each node with maximal support values marked by a black circle. A larger, bold font indicates support for Acoelomorpha/Xenacoela. Branch length scale bars represent substitutions/site.

To compare support for Acoelomorpha and Xenacoela in the new filtered datasets, I assessed the fit of a variety of models and performed phylogenetic analyses under each tested model. The assumption underlying this approach is that as model fit improves, systematic errors and long-branch attraction should be better attenuated [32,53]. To assess relative model fit, I applied a 10-fold Bayesian cross-validation in Phylobayes [54] to a set of seven increasingly complex models: the site-homogeneous (i) Poisson, (ii) LG (the empirical amino acid exchangability matrix of Le and Gascuel [55]) and (iii) LG + F (taking the average equilibrium frequencies inferred from the data, equivalent to fixing the CAT model to a single category/class) + G (fitting sites to 4 discrete gamma categories to allow for variation in exchangability rates across sites), and the site-heterogeneous (iv) LG + C20 (an empirical variant of the CAT model with sites fit to 20 predefined equilibrium frequency classes/categories [56]) + G, (v) LG + C60 (as per C20, but with 60 equilibrium frequency classes/categories [56]) + G, (vi) LG + CAT + G and (vii) GTR + CAT+ G. Relative model fit and complexity correlate across the three datasets, with GTR + CAT + G always in the set of best-fitting models, and joined by LG + CAT + G for Cannon336-X and Marlétaz-X (figure 2*a*).

As noted earlier, compositional heterogeneity across sites and taxa is a major source of systematic error contributing to incongruence in animal and bilaterian phylogenomics [28,29,31,34,39]. To test absolute model fit in the context of compositional heterogeneity for all three datasets, I employed posterior predictive analyses (PPAs) in Phylobayes [34,39,54]. Specifically, I performed tests of the average (PPA-MEAN) and maximum (PPA-MAX) compositional heterogeneity across taxa and of per site amino acid diversity (PPA-DIV) (i.e. compositional heterogeneity across sites [34,39,54]). These tests were applied to the same seven models, and also to Dayhoff6 recoded datasets under GTR + CAT + G. All datasets drastically fail the PPA-DIV test when simple models are used but adequacy appears to correlate with complexity and relative fit, and only Cannon336-X fails (pass range: 2 > |Z| > −2), and then barely, under the site-heterogeneous GTR + CAT + G model (figure 2*b*). For Marlétaz-X, GTR + CAT + G appears to have a slight edge over LG + CAT + G, despite having a similar relative fit (figure 2*b*). Consistent with the removal of sites associated with compositional heterogeneity across taxa, all datasets and modelling approaches pass (at 2 > Z > −2) the PPA-MAX test. However, only Philippe-X passes PPA-MEAN at the amino acid level, while Cannon336-X fails even when recoded (figure 2*c*). Interestingly, this is due to overestimating, rather than underestimating, compositional heterogeneity across taxa, perhaps due to trimming of sites associated with this. These results indicate that compositional heterogeneity can be modelled reasonably well for Philippe-X and Marlétaz-X, but less well for Cannon336-X.

**Figure 3.**
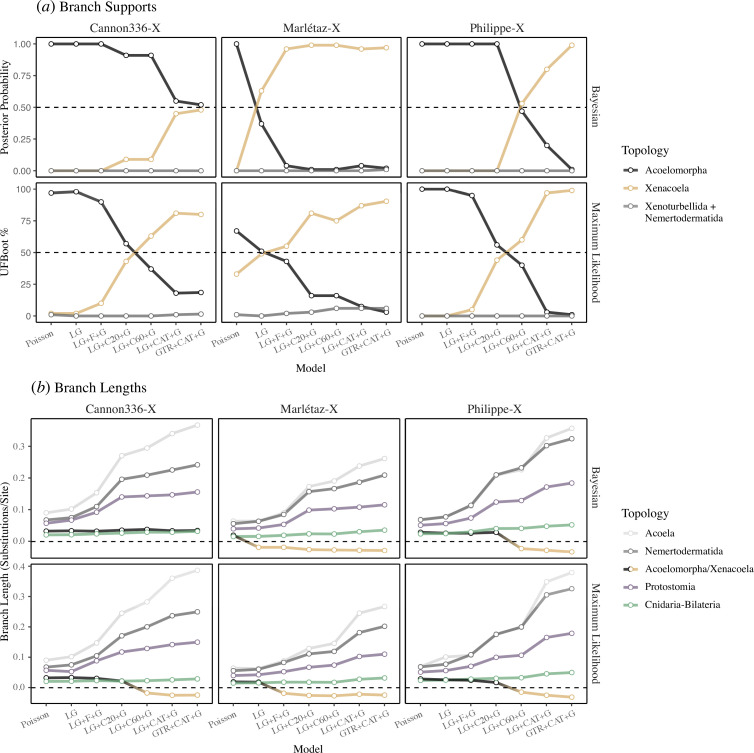
Comparison of branch supports and lengths across models for the Cannon336-X, Marlétaz-X and Philippe-X datasets. (*a*) Posterior probability (Bayesian) and UFBoot (maximum likelihood) support values for the three possible relationships between the major Xenacoelomorpha lineages under the seven amino acid models applied, ordered in accordance with model complexity and fit (though note that LG + CAT + G and GTR + CAT + G fit similarly well for Cannon336-X, and to a lesser extent Marlétax-X). (*b*) Branch lengths across the same seven models for Bayesian and maximum likelihood analyses. The ancestral Acoelomorpha and Xenacoela branches are treated as the same variable (Xenacoela branch lengths are coded as negative values) and plotted as a single line for comparison to how model fit alters the length of ancestral branches of other (control) clades. For maximum likelihood analyses, the mean estimate is used for CAT-PMSF-based analyses (as these models were inferred from two Bayesian chains) for both UFBoot percentage and branch lengths.

Analysing all three datasets under the best-fitting models always recovers maximal support for the monophyly of each of Xenacoelomorpha, Acoela and Nemertodermatida (figure 2*d*). However, Acoelomorpha is only recovered for Cannon336-X, which poses the greatest modelling challenge, and only with equivocal support (posterior probability (PP): LG + CAT+ G = 0.55, GTR + CAT + G = 0.52; figure 2*d*; electronic supplementary material, figure S2). Instead, I find significant support for Xenacoela, with *Xenoturbella* sister to Acoela, in the Philippe-X (PP = 0.99) and Marlétaz-X (PP: LG + CAT + G = 0.96, GTR + CAT+ G = 0.97) analyses (figure 2*d*; electronic supplementary material, figure S2). By comparison, analyses under poorly fitting Poisson recovers Acoelomorpha with maximal support for all three datasets (figure 3*a*; electronic supplementary material, figure S2), but Xenacoela is already strongly supported under site-homogeneous LG + F + G (PP = 0.96) for Marlétaz-X (figure 3*a*; electronic supplementary material, figure S2). The results reveal a clear trend where support for Acoelomorpha decreases and support for Xenacoela increases under progressively better fitting models (figure 3*a*; electronic supplementary material, figure S2). Although the use of recoding is under debate [37–42,57], Dayhoff6 recoding combined with GTR + CAT + G offers the best modelling of compositional heterogeneity (figure 2*b,c*) and supports Xenacoela for all three datasets (electronic supplementary material, figure S2), strongly for Marlétaz-X (PP = 1) and Philippe-X (PP = 0.99) and weakly for Cannon336-X (PP = 0.66).

I also performed maximum likelihood analyses in IQ-TREE [58] under a set of seven comparable models, with the key difference being the use of the posterior mean site frequencies (PMSF) approach for the LG + C20 + G and LG + C60 +G models [59], and the CAT-PMSF approach [60] (which employs site-specific amino acid frequencies and, if using GTR, average amino acid exchangeabilities inferred from Phylobayes) for LG + CAT + G and GTR + CAT + G. Testing the fit of PMSF models is not straightforward [59], however, I contend that the earlier Bayesian model testing offers a reasonably informative guide for these closely corresponding models. The phylogenetic results corroborate the Bayesian analyses, showing a shift away from Acoelomorpha and towards Xenacoela as better fitting models are applied (figure 3*a*; electronic supplementary material, figure S2). Interestingly, unlike the Bayesian analyses, support for Xenacoela overtakes support for Acoelomorpha as better fitting models are applied for Cannon336-X (figure 3*a*; electronic supplementary material, figure S2).

An important consideration given the filtering applied here is the gene content of each dataset. As such, variant datasets using only genes unique to each dataset were also generated, revealing that Xenacoela is recovered under the best-fitting amino acid models for Marlétaz-X and Philippe-X, but only when recoding for Cannon336-X when only genes unique to each dataset are used (electronic supplementary material, figure S2).

Despite site-heterogeneous models suppressing long-branch attraction, their improved detection of hidden substitutions produces trees with longer branches (measured in substitutions/site) [30,33,34]. This means that well-supported clades will often have a longer ancestral branch joining them to the rest of the tree under such models [33]. Consistent with this, the branch leading to Protostomia and the branch splitting Cnidaria and Bilateria grow longer as model fit increases (figure 3*b*). Consistent with the monophyly of each lineage and better detection of hidden substitutions in long-branching or fast-evolving lineages with better fitting site-heterogeneous models, the branches leading to Acoela and Nemertodermatida become far longer as model fit improves (figure 3*b*). Contrarily, but consistent with branch support values, the branch leading to Acoelomorpha becomes shorter as model fit improves, and eventually switches to a branch leading to Xenacoela that lengthens with further improvement of model fit (though with the exception of the Cannon336-X Bayesian analysis; figure 3*b*).

In all, these results suggest that Acoelomorpha is a systematic error, with Xenacoela being recovered when the data are better modelled.

### (d) Simulations implicate Acoelomorpha but not Xenacoela as an error

Simulations allow the propensity for opposing topologies to derive from phylogenetic error to be compared [30,33] by simulating alignments under each topology and testing whether there is an asymmetry in accurate topology recovery when the data are analysed [30]. I applied this approach under the hypothesis that Acoelomorpha would be easily recovered when correct, and that it might also be recovered by long-branch attraction when incorrect.

**Figure 4.**
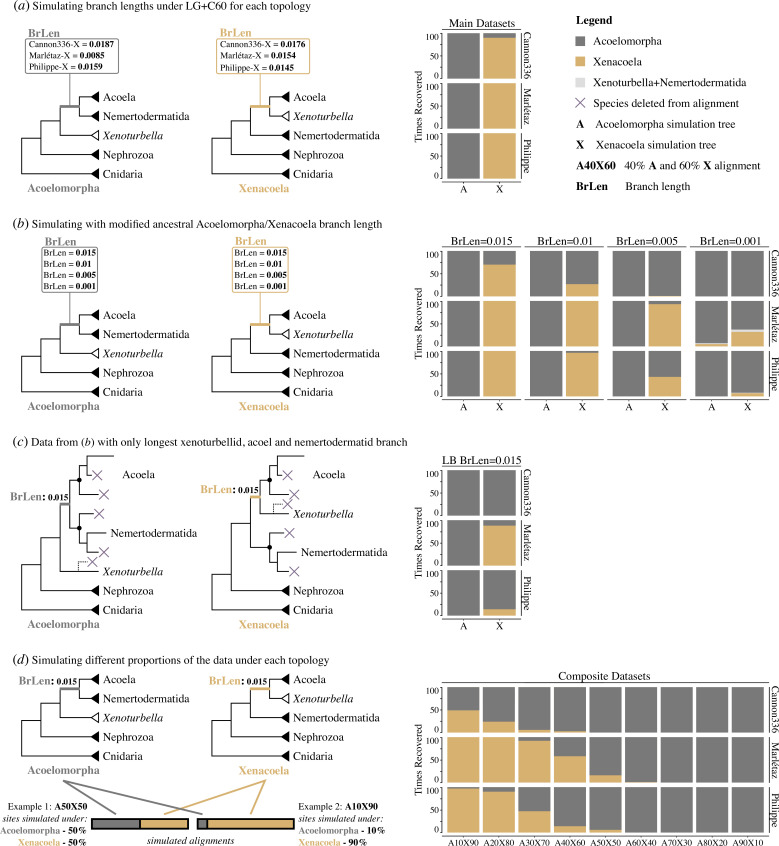
Contrasting recovery frequency of Acoelomorpha and Xenacoela in systematic error-inducing simulations**.** (*a*) Simulations under both LG + F + G topology and a modified topology supporting the alternative Acoelomorpha/Xenacoela topology for the Cannon336-X, Marlétaz-X and Philippe-X datasets with branch lengths inferred under LG + C60 PMSF. The inferred empirical branch length supporting each topology is shown for each dataset alongside bar plots recording the number of times each topology is recovered from 100 simulations when analysed under LG + F + G. (*b*) Simulations and bar plots as per part (*a*) but with modified branch lengths to equalize the length of the ancestral Acoelomorpha/Xenacoela branch. (*c*) Results for the part (*b*) simulations with the longest (BrLen = 0.015) ancestral Acoelomorpha/Xenacoela branch when all but the longest branching species from *Xenoturbella*, Acoela and Nemertodermatida are excluded. (*d*) Simulations on the trees from part (*b*) with the longest ancestral Acoelomorpha/Xenacoela branch (BrLen = 0.015) but with different proportions of the alignment simulated under each tree topology rather than all under a single topology, as well as bar plots of topology recovery count. Nephrozoa is shown as a clade to simplify presentation but is not always present in the simulating trees. The *Xenoturbella* clade is shown in white in parts (*a*), (*b*) and (*d*), whilea dotted line shows removed *Xenoturbella* species in part (*c*), as only Marlétaz-X contains more than one species.

First, taking the basic LG + F + G tree topologies (but with branch lengths estimated under LG + C60 PMSF) for each dataset, and a modified version with the alternative topology (Acoelomorpha/Xenacoela), I simulated 100 alignments of 25 000 sites under the LG + C60 PMSF model for each topology [59,61,62]. Analysing these alignments under the simpler LG + F + G model, which should accentuate the potential for systematic error [30], I always recovered the correct topologies except for a small number of Cannon336-X simulations recovering Acoelomorpha when Xenacoela is true (figure 4*a*), providing little evidence for any clear bias.

However, the length of the ancestral branch leading to Acoelomorpha/Xenacoela is not equal across topologies, meaning the simulation itself is asymmetric, while these branch lengths also likely favour Xenacoela compared with simulating from trees inferred from the unfiltered original datasets. To better explore how ancestral Acoelomorpha/Xenacoela branch lengths influence topology recovery, I modified the simulating trees to remove ancestral branch length asymmetry between topologies (figure 4*b*). Testing the length of this branch at 0.015, 0.01, 0.005 and 0.001 substitutions/site revealed a very clear pattern that cannot be explained by differences in the simulation tree beyond the Acoelomorpha/Xenacoela branch length. Acoelomorpha is almost always recovered when correct, even when the ancestral branch is very short (figure 4*b*). The only exception to this is at the shortest ancestral branch length for Marlétaz-X, where Acoelomorpha recovery drops to 93%. Conversely, Xenacoela recovery is sensitive to ancestral branch length, with erroneous Acoelomorpha recovery at 63–100% when the ancestral branch is at the shortest length (figure 4*b*).

To complement this, I reanalysed the 0.015 substitutions/site ancestral branch alignments with all but the fastest evolving acoel, nemertodermatid, and for Marlétaz-X (which has two xenoturbellids), xenoturbellid, as this should increase the potential for long-branch attraction errors [33] (figure 4*c*). Here, as expected, Acoelomorpha was always recovered when correct, and often erroneously recovered (Cannon336-X = 100%, Marlétaz-X = 12% and Philippe-X = 86%) when Xenacoela was correct (figure 4*c*).

To help understand the influence that orthology errors (and other biological factors such as incomplete lineage sorting or gene flow) might have on topology recovery, I reperformed simulations with 0.015 substitutions/site on the ancestral Acoelomorpha/Xenacoela branch, but varying proportions of the data that were simulated under each tree [30] (figure 4*d*). I tested nine data composition variants spanning 10% windows from 10% Acoelomorpha and 90% Xenacoela to 90% Acoelomorpha and 10% Xenacoela (figure 4*d*). Unbiased results can be expected to record either topology in approximately 50% of cases (and likely with weak support) when 50% of the data are simulated under each topology [32]. However, I observe a clear bias in favour of Acoelomorpha, which is always recovered (except for a single tree with 60% simulated under Acoelomorpha for Marlétaz-X) when it is the majority simulation tree and is always the most frequently recovered topology when 50% of the data are simulated under each topology (figure 4*d*). Conversely, even when 90% of the data are simulated under Xenacoela, Acoelomorpha is still recovered in some cases, and in most cases for Cannon336-X (figure 4*d*).

These simulations highlight the influence of branch length, an important factor influencing branching order in animal phylogenomics [30,34,57,63], on simulation outcomes, and clearly indicate that Acoelomorpha: (i) is more easily recovered than Xenacoela when correct and (ii) is easily recovered in error when Xenacoela is correct.

### (e) Support for Nephrozoa is strongest under poorly fitting models

**Figure 5.**
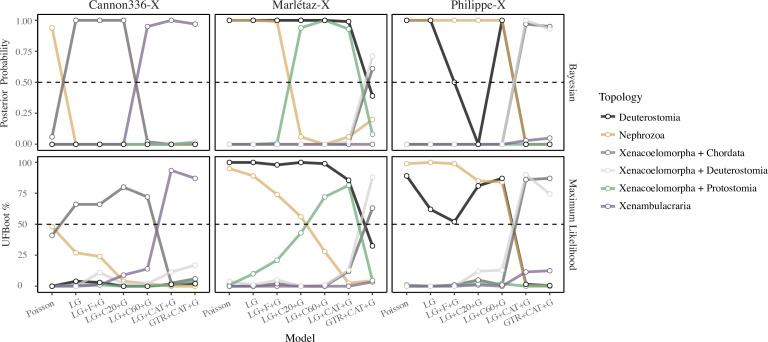
Comparison of branch supports for the different placements of Xenacoelomorpha amongst Bilateria and for deuterostome monophyly across models for the Cannon336-X, Marlétaz-X and Philippe-X datasets. Posterior probability (Bayesian) and UFBoot (maximum likelihood) support values for different relationships under the seven amino acid models applied, ordered in accordance with model complexity and fit (though note that LG + CAT + G and GTR + CAT + G fit similarly well for Cannon336-X, and to a lesser extent Marlétax-X). For maximum likelihood analyses the mean estimate is used for CAT-PMSF-based analyses (as these models were inferred from two Bayesian chains). ‘Deuterostomia’ alone refers to Ambulacraria + Chordata, such that Xenacoelomorph + Ambulacraria or Xenacoelomorpha + Chordata are excluded but not Xenacoelomorpha sister to both, while all three placements are included in ‘Xenacoelomorpha + Deuterostomia’.

Placing Xenacoelomorpha in the bilaterian tree of life is among the most vexing problems in animal phylogenomics [18,25,28,29]. The datasets used here are small and internal Xenacoelomorpha targeted, meaning their validity for testing the placement of Xenacoelomorpha is not entirely clear. Nevertheless, a general pattern of support for Nephrozoa being suppressed as better-fitting models are applied can be observed (figure 5; electronic supplementary material, figure S2), consistent with recent reports [28–31]. However, unlike the shift observed from Acoelomorpha towards Xenacoela, the primary alternative hypothesis, Xenambulacraria [3,28–30], does not clearly or consistently emerge under better modelling conditions (figure 5; electronic supplementary material, figure S2). For Cannon336-X, Xenambulacraria replaces Nephrozoa, with an unexpected intermediate grouping of Xenacoelomorpha + Chordata, as better fitting models are applied (figure 5; electronic supplementary material, figure S2), but strong support is lost with recoding (electronic supplementary material, figure S2). Marlétaz-X begins shifting towards Xenacoelomorpha + Protostomia as model fit improves, but Xenacoelomorpha + Chordata is recovered under GTR + CAT + G (figure 5; electronic supplementary material, figure S2), and also when using either only genes unique to the dataset or when using only slowly evolving *Xenoturbella* to represent Xenacoelomorpha, which is purported to help avoid systematic error [28] (electronic supplementary material, figure S2). Philippe-X shifts towards Xenacoelomorpha + Chordata as model fit improves (figure 5; electronic supplementary material, figure S2), though recoding shifts weakly towards Xenambulacraria, while Bayesian analyses using only genes unique to this dataset recover Nephrozoa in amino acid analysis (electronic supplementary material, figure S2). In all, these findings do not resolve the sister group to Xenacoelomorpha, but are generally consistent with suggestions that Nephrozoa is a systematic error [28–30].

The monophyly of deuterostomes has also been questioned lately [28,33]. In line with this, regardless of whether Xenacoelomorpha is included, Deuterostomia is not recovered in all datasets or under all models in the analyses performed here. The monophyly of Ambulacraria and Chordata (i.e. Deuterostomia excluding Xenacoelomorpha or with Xenacoelomorpha as sister to these lineages) appears weakly, though not consistently, linked with less well-fitting models (figure 5; electronic supplementary material, figure S2).

## 3. Discussion

The phylogenetic placement of the three xenacoelomorph lineages has been a long-standing problem in evolutionary biology [4,18,21,28–30,64], with *Xenoturbella* having been described as the ‘champion wanderer’ of bilaterian phylogeny [21]. This study sets *Xenoturbella* on its way once more, nesting it deeper within Xenacoelomorpha as the sister group to Acoela. The proposed name for this clade, Xenacoela, is consistent with the naming of Xenacoelomorpha [3] and Xenambulacraria [12], and does not rely on new interpretations of morphological character history. I suggest retaining Xenacoelomorpha as the phylum name, rather than including Xenoturbellida within Acoelomorpha, as this maintains coherence with Xenambulacraria [12]. I propose that Xenacoela should take the place of Acoelomorpha, which appears to be invalid, as a subphylum to Xenacoelomorpha. If accepted, this implies the additional taxonomic revisions of demoting Xenoturbellida to class alongside Acoela and raising Nemertodermatida to subphylum.

My results suggest that the monophyly of the acoelomorph flatworms derives from long-branch attraction between the fast-evolving Acoela and Nemertodermatida. Looking beyond this, it might be questioned whether the remarkably long branches of Acoela also exclude *Xenoturbella* through further, as yet undetectable, long-branch attraction. However, this seems unlikely, as the branch leading to Acoela becomes dramatically longer as model fit improves (figure 3*b*), lending confidence to the monophyly of Acoela.

The monophyly of Acoelomorpha has been questioned in the past [24,65–67]. However, this predated the discovery of Xenacoelomorpha and specifically referred to Nemertodermatida being sister to Nephrozoa, with Acoela sister to both, implying an acoelomorph flatworm-like bilaterian ancestor [24,65,66]. The findings here are distinct as: (i) Xenacoelomorpha is unsurprisingly (only genes recovering this clan were used) recovered as monophyletic, (ii) the non-monophyly of Acoelomorpha is with respect to *Xenoturbella* rather than Nephrozoa, and (iii) when placing the results here in the context of recent studies [28–30], Nephrozoa appears likely to be a systematic error, which is at odds with an acoelomorph-like last common bilaterian ancestor.

That I find Acoela and Nemertodermatida to cause long-branch attraction even within Xenacoelomorpha might be taken to provide auxiliary evidence for long-branch attraction to affect their placement amongst Bilateria [3,28–30]. This may lend credence to past analyses using only slow-evolving *Xenoturbella* to represent all of Xenacoelomorpha, where support leans towards Xenambulacraria [28], though this was not the case across datasets here (electronic supplementary material, figure S2). Consistent with recent studies [28–32], I find that support for Nephrozoa is typically associated with simple, poorly fitting models. However, I do not recover a consistently strong signal for Xenacoelomorpha’s closest relatives across datasets under the best-fitting models. Similarly, and again in line with recent studies [28,31,33], deuterostome monophyly was not clearly supported across the analyses performed here. Notably, the datasets used here are dramatically reduced compared with the original counterparts [18,28,31], and only include genes recovering Xenacoelomorpha as a clan in gene trees. While these genes could be argued to produce a more coherent signal for the relationships between Xenacoelomorpha and other animals, I did not similarly consider the recovery of other clans beyond Xenacoelomorpha [29]. Rather than solving these issues, the results clearly highlight the difficulty of resolving deep bilaterian relationships due to phylogenomic sensitivity to data and model choices.

Many questions about gene choice, dataset trimming, phylogenetic modelling approaches, recoding and their intersection remain open and debated in phylogenomics [29,32,34,37,44,60,68–76]. I am, nonetheless, optimistic that strategies like that applied here can provide a path forward for the detection and resolution of previously hidden long-branch attraction artefacts across the tree of life. Pairing stringent, focused data filtering and well-fitting models should help to minimize branching artefacts by improving the signal-to-noise ratio. The associated reduction in computational requirements makes phylogenomic analyses more environmentally friendly [77], accessible and reproducible.

Importantly, Xenacoela recovery is not restricted to a single set of strict conditions. For example, different subsampling of genes [29] or the combined application of site-heterogeneous models and recoding to large phylogenomic datasets (>1000 genes) [28], have both recovered Xenacoela in previous studies (figure 1*d*). Xenacoela also joins the shortest and longest branching xenacoelomorph lineages, instead of the two longest branching lineages (as Acoelomorpha does) as might be expected in the case of long-branch attraction. The simulations performed here support this, showing that Acoelomorpha is almost always recovered when it is the correct tree and is also easily recovered in error, whereas Xenacoela is more difficult to recover when correct and is only erroneously recovered in extreme cases and then rarely.

In summary, my results reject Acoelomorpha in favour of Xenacoela and indicate that Nephrozoa is likely a systematic error. Chromosome-scale genome sequences from across Xenacoelomorpha will allow further comparison of support for Xenacoela or Acoelomorpha through syntenic evidence producing large, reliable orthologue sets and potentially informative rare genomic changes [78–81]. I predict that careful taxonomic consideration of Xenacoela will reveal morphological characters that represent synapomorphies uniting *Xenoturbella* and Acoela, as very few morphological characters unite Acoelomorpha [82], and this has not been reappraised since the establishment of Xenacoelomorpha.

## 4. Methods

See the electronic supplementary material for details of the methods used in this study [83].

## Data Availability

Datasets, alignments, tree files, model fit statistics and simulation data and trees are available on Figshare at [84]. Supplementary material is available online [83].
